# FilmArray® Meningoencephalitis panel in the diagnosis of central nervous system infections: stewardship and cost analysis in a paediatric hospital in Chile

**DOI:** 10.1186/s12887-022-03241-1

**Published:** 2022-04-05

**Authors:** Mirta Acuña, Dona Benadof, Karla Yohannessen, Yennybeth Leiva, Pascal Clement

**Affiliations:** 1 Pediatric Infectious Disease Department, Roberto del Río Children’s Hospital, Av. Profesor Zañartu 1085, Independencia, Santiago, Chile; 2 Department of Pediatrics and Pediatric Surgery, Facultad de Medicina, Universidad de Chile, Av. Independencia 1027, Independencia, Santiago, Chile; 3 Laboratory Department, Roberto del Río Children’s Hospital, Av. Profesor Zañartu 1085, Independencia, Santiago, Chile

**Keywords:** Diagnostic stewardship, CNS infections, FilmArray®, Children

## Abstract

**Background:**

Central nervous system (CNS) infection has been an ongoing concern in paediatrics. The FilmArray® Meningoencephalitis (FAME) panel has greater sensitivity in identifying the aetiology of CNS infections. This study’s objective was to compare the aetiological identification and hospitalization costs among patients with suspected CNS infection before and after the use of FAME.

**Methods:**

An analytical observational study was carried out using a retrospective cohort for the pre-intervention (pre-FAME use) period and a prospective cohort for the post-intervention (post-FAME use) period in children with suspected CNS infection.

**Results:**

A total of 409 CSF samples were analysed, 297 pre-intervention and 112 post-intervention. In the pre-intervention period, a total of 85.5% of patients required hospitalization, and in the post-intervention period 92.7% required hospitalization (*p* < 0.05). Median of ICU days was significantly lower in the post-intervention period than it was in the pre-intervention period. The overall positivity was 9.4 and 26.8%, respectively (*p* < 0.001). At ages 6 months and below, we found an increase in overall positivity from 2.6 to 28.1%, along with an increased detection of viral agents, *S. agalactiae, S. pneumoniae*, *and N. meningitidis.* The use of this diagnostic technology saved between $2916 and $12,240 USD in the cost of ICU bed-days. FAME use provided the opportunity for more accurate aetiological diagnosis of the infections and thus the provision of adequate appropriate treatment.

**Conclusions:**

The cost/benefit ratio between FAME cost and ICU bed-day cost savings is favourable. Implementation of FAME in Chilean public hospitals saves public resources and improves the accuracy of aetiological diagnosis.

## Introduction

Central nervous system (CNS) infections have been an ongoing concern in paediatrics due to the burden of disease, the risk of severe neurological sequelae, and the high mortality rate associated mainly with bacterial aetiologies. For this reason, the challenge over decades has been to improve the aetiological diagnosis of CNS infections, to shorten the window of opportunity during which this is performed, and to optimize the clinical management and outcomes of patients.

A few decades ago, bacterial aetiologies were the most common, especially *H. influenzae* and *S. pneumoniae* [1, 2]. However, since the introduction of the *H. influenzae* b and pneumococcal conjugate vaccine, the epidemiology of meningitis has changed [3]. Currently, most cases of meningitis are caused by viruses (aseptic meningitis) [4–6]. Nigrovic et al., in a multicentre retrospective cohort (*n* = 3295) of patients between 1 month and 18 years of age, found that 96.3% of cases were aseptic meningitis (culture or latex negative for bacteria), while only 3.7% of cases were caused by a bacterial agent [7]. Among the most common bacterial pathogens in infants under 3 months of age are *Streptococcus agalactiae* and gram-negative rods, while in older infants, bacterial meningitis is caused mainly by *S. pneumoniae* of non-vaccine serotypes and *N. meningitidis* [4, 8]. In Chile, the vaccine for *H. influenzae* type b was incorporated into the National Vaccine Program in 1996, that for *S. pneumoniae* was incorporated in 2010, and that for *N. meningitidis* serotypes A, C, W, and Y was incorporated in 2012; together these measures sought to reduce the incidence of acute bacterial meningitis (ABM) due to these pathogens.

In 2011, in Roberto del Río Children’s Hospital, 73 cerebrospinal fluid (CSF) samples with altered cytochemical characteristics were classified as meningitis or meningoencephalitis; of these, 28.7% were suggestive of a bacterial pattern and the rest were consistent with aseptic meningitis (unpublished data).

Molecular diagnostic techniques have progressed rapidly in recent years, leading to modern techniques of diagnosing CNS infectious disease [9]. At present, the diagnosis of viral CNS infections is made using polymerase chain reaction (PCR) techniques, resulting in the aetiological confirmation of infection source in up to 45% of cases [10]. FilmArray® Meningoencephalitis panel (FAME) technology analyses CSF and is based on the specific amplification of certain regions of the pathogen genome using multiple nested PCRs. It presents a good correlation with conventional PCR, in addition to having a greater sensitivity in the detection of co-infections and a greater likelihood of identifying the aetiology of the infection [11–13]. This technology may also impact the cost effectiveness of treatment interventions due to the better stewardship of resources secondary to accurate aetiological identification of infectious agents [14]. Faced with this, we posed the following question: Does the use of FAME improve the aetiological identification of infectious agents and reduce hospitalization costs among patients with suspected CNS infection treated at the Roberto Del Río Children’s Hospital? The main objective of the study was to compare the aetiology of the infection source among patients with suspected CNS infection with their associated hospitalization costs both before and after the clinical use of FAME.

## Methods

### Study design

An analytical observational study was carried out, before and after the clinical application of FAME, using a retrospective cohort for prior to the intervention and a prospective cohort for post-intervention period. This period was defined as a pre-intervention of 12 months (January to December 2016) and post-intervention period of 14 months (January 2017 to February 2018). The difference of 2 months between periods, lies in the availability of the FAME kit.

We worked with the entire range of children in whom CNS infection was suspected according to the criteria described below.

### Population

The study was conducted at the Roberto Del Río Children’s Hospital, which is the only public paediatric hospital belonging to the North Metropolitan Health Service in Santiago, Chile. It is a highly complex institution, with an assigned paediatric population of approximately 200,000 children under 15 years of age according to the 2017 national census [15]. The population study included outpatient, inpatient, and patients seen in the emergency department (ED), which operates 24/7. In this establishment, each patient in whom a CNS infection was suspected routinely underwent a lumbar puncture, from which 3 samples were obtained: one for culture and gram stain, one for cytochemical analysis, and one critical sample for molecular biology.

### Inclusion and exclusion criteria

The following inclusion criteria were considered for the present study: (I) patients under 15 years of age; (II) patients with at least one of the following independent conditions: (a) pleocytosis: > 10 cells/μl after the neonatal period (28 days) or > 20 cell/μl in children under 1 month [16, 17]; (b) age < 3 months with a recent story of fever or documented fever at the emergency room; or (c) previously healthy patients with focal neurological signs or compromised consciousness not explained by trauma or intoxication; and (III) only the first sample from each patient that met the above criteria was used.

The exclusion criteria were (I) insufficient amount of CSF, (II) inadequate sample, or (III) samples derived from neurological, neurosurgical, or oncological patients.

### Laboratory

Samples were processed in the clinical laboratory of Roberto del Río Children’s Hospital. During 2016, CSF microbiological studies were carried out by culturing on Columbia agar + 5% sheep’s blood, chocolate agar, and MacConkey agar plates, carrying out their identification and susceptibility according to the Clinical and Laboratory Standards Institute (CLSI) standards. This result was available within 48 h.

In addition, real-time PCR was performed for bacteria (*S. pneumoniae*, *N. meningitidis*, *H. influenzae*), and real-time PCR was performed for viruses (herpes simplex 1 and 2, enterovirus, Epstein Barr, cytomegalovirus, human herpes 6). The PCRs were requested at the discretion of the treating medical teams. The turnaround time for these tests is 24 to 48 working hours in our molecular laboratory.

Since 2017, in addition to the techniques described above, the FAME panel has been incorporated into the microbiological analysis of CSF. This technique identifies 14 neurotropic pathogens, including 6 bacteria: *E. coli* K1, *H. influenzae*, *L. monocytogenes*, *N. meningitidis*, *S. agalactiae* and *S. pneumoniae*; 7 viruses: cytomegalovirus (CMV), enterovirus, herpes simplex virus 1 (HSV-1), herpes simplex virus 2 (HSV-2), human herpes virus 6 (HHV-6), human parechovirus (HPeV), varicella zoster virus (VZV), and Epstein Barr virus (EBV); and a fungus: *Cryptococcus neoformans/gattii*. This technology automatically extracts DNA and processes the PCRs in a closed system, with a turnaround time of 65 min [12, 18, 19]. During the post-intervention study period, samples were processed 24/7 in the emergency laboratory.

### Data source

The subject’s data were obtained from the electronic clinical record and laboratory data management system (REAL®). The independent variable (exposure) was the use of the FAME diagnostic technique, and the covariables were age, sex, CSF cytochemical results, CSF microbiological result, discharge diagnosis, outpatient treatment or hospitalized management. Dependent variables included days in hospital beds and type of bed complexity (ICU/basic bed), antimicrobial use and performance of complementary diagnostic studies (brain CT, brain MRI and EEG).

### Costs

For cost analysis, we decided to focus primarily on cost per bed day. For this, the costs of a basic bed day and an ICU bed day in the public and private health systems were used, and their costs were obtained from the standardized fees of the public health system and the average costs from 5 private institutions in the metropolitan region of Santiago, Chile.

The indicators used comparatively were absolute numbers of bed-days, bed-days used per patient and percentiles of bed-day use. We had difficulties in assessing the costs of antimicrobial treatments, so we decided to exclude them from this analysis. Routine laboratory testing costs was not analysed due to standardized hospital protocols in suspected CNS infection, all patients received the same laboratory exploration, so it is not a variable. The current currency used was US dollars to facilitate universal understanding, although the Chilean currency is “Chilean pesos” (Parity of chilean peso (CLP) $801.96 per dollar at 16 November 2021) [20].

### Statistical analysis

An exploratory analysis of the data was performed to find and correct anomalous or duplicate values ​​and to study the distribution of quantitative variables. Descriptive statistics were used to report the general and clinical characteristics of the patients, the results of the microbiological examinations, the performance of imaging studies and the performance of the microbiological study on the analysed CSF samples. Position measures were reported for continuous variables due to their nonsymmetric distribution, and absolute and relative frequencies (%) were reported for categorical variables. All the sets of variables mentioned were compared between the study periods using the chi-square test, Fisher’s exact test, or the two-sample test of proportions, in the case of categorical variables according to the fulfilment of assumptions. The comparison of the quantitative variables between both periods was calculated through the Mann–Whitney test. The level of significance for all the analyses described was established at *p* < 0.05. Statistical analyses were performed using Stata 13 SE software.

## Results

**Fig. 1:**
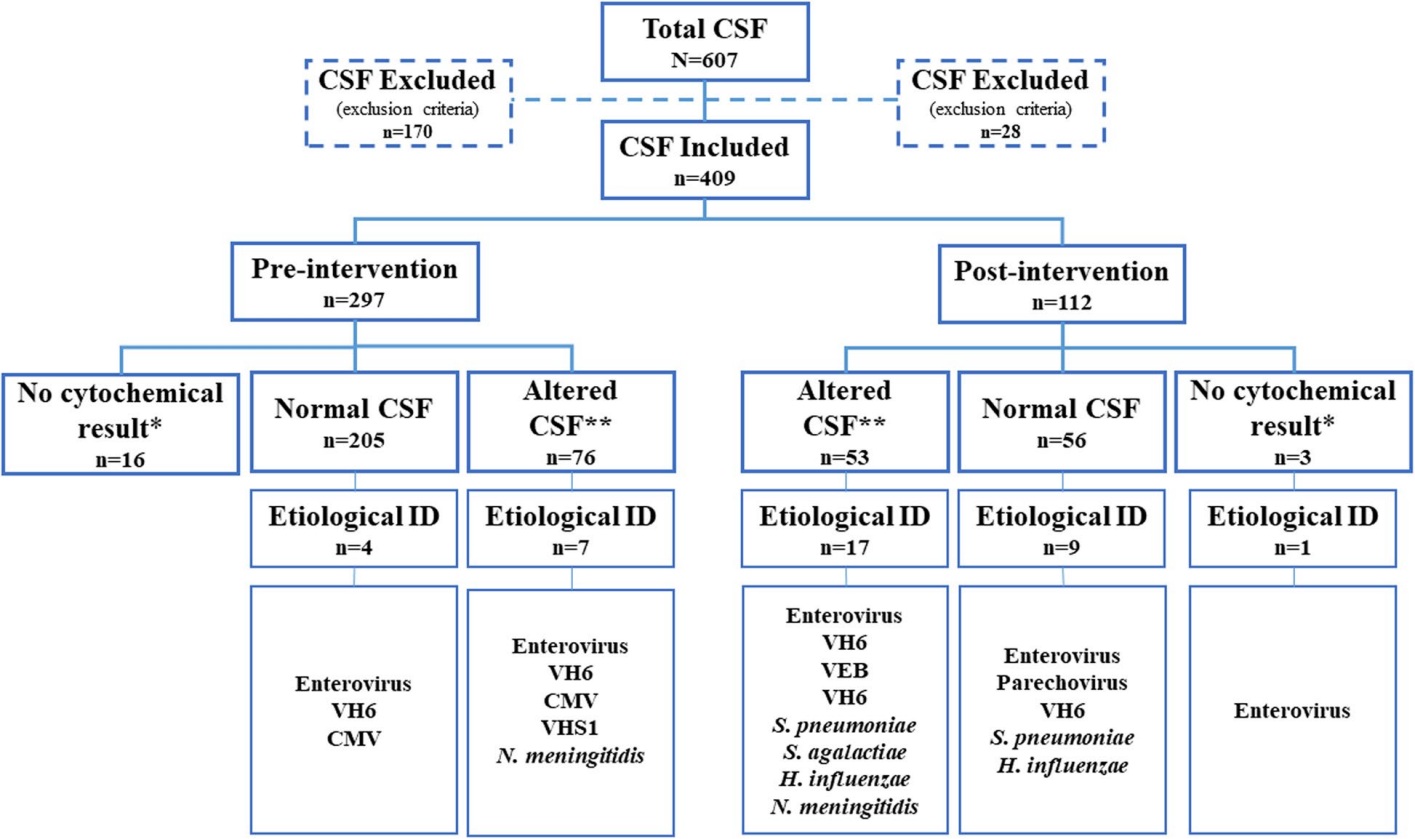
Patients included and aetiological identification of infectious agents. **Altered CSF: > 10 cells in children older than 1 month; > 20 cells in children under 1 month. *No cytochemical result: coagulated, traumatic, scarce samples, no data

During the study period, 607 CSF samples were obtained from children under 15 years of age at the Roberto del Río Children’s Hospital. A total of 198 were excluded, and 409 CSF samples were finally analysed for CNS infection (Fig. 1). Of these samples, 297 were collected during the pre-intervention period and 112 were collected during the post-intervention period. Of these, 39.7 and 46.4%, respectively, were female. Median of age of the subjects studied was 1.6 (IQR: 0.8-12.7) and 2.4 (IQR: 1.0-28.7) months for the retrospective and prospective cohorts, respectively. Age was stratified into 4 groups: < 6 months, 6-23 months, 24-71 months, and > 72 months (Table 1).

**Table 1:** General and clinical characteristics of the patients studied

	Pre-intervention ***n*** = 297	Post-intervention ***n*** = 112	***p*** value
**Female, n (%)**	118 (39.7%)	52 (46.4%)	0.220^a^
**Age (month), Median (IQR)**	1.6 (0.8-12.7)	2.4 (1.0-28.7)	0.043^b^
**Age categories**			
< 6 months	195 (65.7%)	64 (57.1%)	0.085^a^
6- < 24 months	50 (16.8%)	16 (14.3%)	
24 - < 72 months	27 (9.1%)	19 (17.0%)	
> = 72 months	25 (8.4%)	13 (11.6%)	
**Discharge diagnosis, n (%)**			
Other focus no CNS of infectious diseases	76 (25.6%)	11 (9.8%)	**< 0.001**^**c**^
Fever without focus or prolonged	89 (29.9%)	22 (19.6%)	**0.018**^**c**^
Seizures and epilepsy	54 (18.2%)	25 (22.3%)	0.168 ^**c**^
Ataxia and rhomboencephalitis	1 (0.34%)	6 (5.4%)	**< 0.001**^**c**^
CNS infectious	41 (13.8%)	34 (30.4%)	**< 0.001**^**c**^
Others	36 (12.1%)	14 (12.5%)	0.456 ^**c**^
**Pleocitosis (≥10 white cells in CSF), n (%)**	85 (30.3%)^d^	52 (47.7%)^d^	**0.001ª**
**Hospitalization requirement, n (%)**	254 (85.5%)	104 (92.7%)	**0.045ª**
**Hospitalization days, Median (IQR)**	4 (2-6)	4 (2-7)	0.485^b^
**ICU requirement, n (%)**	44 (14.8%)	32 (28.6%)	**< 0.001ª**
**ICU days, Median (IQR)**	3.5 (2-7.5)	2 (1-4)	**0.043**^**b**^
**Use of ATB on admission, n (%)**	187 (63.4%)^d^	68 (61.3%)^d^	0.692^a^
**Use of ATV on admission, n (%)**	30 (10.1%)	28 (25.0%)	**< 0.001**^a^
**TAC performed, n (%)**	64 (21.5%)	38 (33.9%)	**0.010**^a^
**RNM performed, n (%)**	33 (11.1%)	21 (18.7%)	**0.042**^a^
**EEG performed, n (%)**	74 (24.9%)	44 (39.3%)	**0.004**^a^

^a^chi-square test

^b^Mann–Whitney test

^c^two-sample test of proportions

^d^Missing data for “Pleocitosis” pre-intervention *n* = 17 and post-intervention *n* = 3 and missing data for “Use of ATB on admission” pre-intervention *n* = 2 and post-intervention *n* = 1; in bold significant *p* values; *ATB* antibiotics, *ATV* antivirals

Of the included patients, 85.5% required hospitalization in the pre-intervention period, compared with 92.7% in the post-intervention period (*p* < 0.05). Of these, hospitalization in the ICU was more frequent in the post-intervention period (14.8% vs. 28.6% (*p* < 0.001)). However, median of ICU bed-days used was significantly lower in the post-intervention period (2 days) than in the pre-intervention period (3.5 days). ICU admission criteria were based on the Paediatric Index of Mortality (PIM), which is an indicator of paediatric severity validated for children under 16 years of age, which is measured when patients are admitted to hospital critical units and whose score ranges from 0 (no risk of mortality) to 100 (predicts certain mortality) [21]. In both groups, the median PIM score was 1.2 (IQR: 0.6-2.8), with no statistically significant differences between the two groups (*p* = 0.266, for the Mann-Whitney test, data not shown). This complements the results of the comparison of ICU bed days between both periods, making them comparable in terms of severity parameters.

There was no significant difference in the use of antibiotics upon admission to the hospital, but there was a difference in the use of antivirals, with a greater use recorded in the post-intervention period (*p* < 0.001) (Table 1). This is probably related to an increase in the aetiological identification of viral infectious agents, but establishing this association was not one of the objectives of this study.

The complementary diagnosis in patients who were hospitalized was significantly higher in the post-intervention period. However, the most frequent discharge diagnoses in the post-intervention period were CNS infections, seizures and epilepsy (all related to the CNS), unlike the diagnoses from the pre-intervention period, which were most frequently fever without an identified source and infection; both were statistically significant (Table 2).

**Table 2:** Diagnostic and complementary studies

	Pre-intervention ***n*** = 297	Post-intervention ***n*** = 112	***p*** value^a^
**TAC performed**	**64**	**38**	
**Diagnosis, n (%)**			
Seizures and epilepsy	34 (53.1%)	14 (36.8%)	0.122
CNS infectious	14 (21.9%)	8 (21.0%)	
Other focus no CNS of infectious diseases	4 (6.3%)	2 (5.3%)	
Fever without focus or prolonged	3 (4.7%)	2 (5.3%)	
Ataxia and rhombencephalitis	1 (1.5%)	6 (15.8%)	
Others	8 (12.5%)	6 (15.8%)	
**RNM performed**	**33**	**21**	
**Diagnosis, n (%)**			
Seizures and epilepsy	15 (45.5%)	3 (14.2%)	**0.025**
CNS infectious	11 (33.3%)	9 (42.9%)	
Other focus no CNS of infectious diseases	1 (3.0%)	–	
Fever without focus or prolonged	–	1 (4.8%)	
Ataxia and rhombencephalitis	1 (3.0%)	5 (23.8%)	
Others	5 (15.2%)	3 (14.3%)	
**EEG performed**	**74**	**44**	
**Diagnosis, n (%)**			
Seizures and epilepsy	40 (54.0%)	19 (43.2%)	0.350
CNS infectious	14 (18.9%)	11 (25.0%)	
Other focus no CNS of infectious diseases	5 (6.8%)	3 (6.8%)	
Fever without focus or prolonged	6 (8.1%)	1 (2.3%)	
Ataxia and rhombencephalitis	1 (1.4%)	3 (6.8)	
Others	8 (10.8%)	7 (15.9%)	

^a^Fisher’s exact test; in bold significant *p* values

### Microbiology

With respect to the results obtained by conventional microbiology, the Gram stain of the CSF was positive in 1% of patients in the pre-intervention period and positive in 2.7% of patients in the post-intervention period. CSF cultures were positive in 0.7 and 1.8% of patients, respectively. Blood cultures were positive in 5 and 5.3% of patients, respectively. None of the three parameters evaluated, Gram stain, CSF cultures or blood cultures, presented significant differences between the evaluated periods. In the pre-intervention period, the molecular results showed a viral aetiology in 3.4% of the CSF samples analysed. However, in the post-intervention period, positivity increased to 16.07%, identifying viruses with *p* < 0.05 (Table 3).

**Table 3:** Microbiological results

Laboratory tests	Pre-intervention ***n*** = 297	Post-intervention ***n*** = 112	***p*** value^**a**^
**Gram, n (%)**			
**With bacteria**	**3 (1.0%)**	**3 (2.7%)**	0.380
**Without bacteria**	**293 (98.7%)**	**109 (97.3%)**	
**No data**	**1 (0.3%)**	**–**	
**Culture, n (%)**			
**Positive**	**1 (0.3%)**	**2 (1.8%)**	0.126
*S. pneumoniae*	*–*	*1 (50%)*	
*N. meningitidis*	*1 (100%)*	*–*	
*S. agalactiae*	*–*	*1 (50%)*	
**Negative**	**296 (99.7%)**	**110 (98.2%)**	
**Blood culture, n (%)**			
**Positive**	**15 (5.1%)**	**6 (5.4%)**	0.289
*S. pneumoniae*	*–*	*1 (16.7%)*	
*N. meningitidis*	*1 (6.7%)*	*1 (16.7%)*	
*S. agalactiae*	*2 (13.3%)*	*3 (50%)*	
*E. coli*	*2 (13.3%)*	*1 (16.6%)*	
*S. epidermis*	*3 (20.0%)*	*–*	
*S. aureus*	*4 (26.6%)*	*–*	
*S. parasanguinis*	*1 (6.7%)*	*–*	
*E. faecalis*	*1 (6.7%)*	*–*	
*S. pyogenes*	*1 (6.7%)*	*–*	
**Negative**	**274 (92.3%)**	**101 (90.2%)**	
**Unrealized-No data**	**8 (2.7%)**	**5 (4.5%)**	
**Molecular test**^**b**^**, n (%)**			
**Positive**	**10 (3.4%)**	**27 (24.1%)**	**< 0.001**
Enterovirus	*3 (30%)*	*14 (51.9%)*	
Parechovirus	*–*	*3 (11.1%)*	
HSV1	*1 (10%)*	*–*	
VZV	*4 (40%)*	*–*	
CMV	*2 (20%)*	*–*	
HHV-6	*–*	*3 (11.1%)*	
*S. pneumoniae*	*–*	*4 (14.8%)*	
*N. meningitidis*	*–*	*1 (3.7%)*	
*H. influenzae*	***–***	*1 (3.7%)*	
*S. agalactiae*	*–*	*1 (3.7%)*	
**Negative**	**31 (10.4%)**	**86 (76.8%)**	
**Unrealized**	**256 (86.2%)**	**–**	
**Overall**^**c**^**, n (%)**			
**Positive**	**28 (9.4%)**	**30 (26.8%)**	**< 0.001**
**Negative**	**29 (9.4%)**	**82 (73.2%)**	
**Unrealized**	**241 (81.1%)**	**–**	
**Altered LCR, n (%)**	**76 (25.6)**	**53 (47.3)**	**< 0.001**
*Positivity in altered LCR, n (%)*	*7 (9.2%)*	*17 (32.1%)*	**0.001**

^a^chi-square test

^b^2016 only PCR vs. 2017-2018 PCR plus FAME

^c^any microbiological test; Italics: relative frequencies, refers to the previous category in bold; bold: significant *p* values

**Fig. 2:**
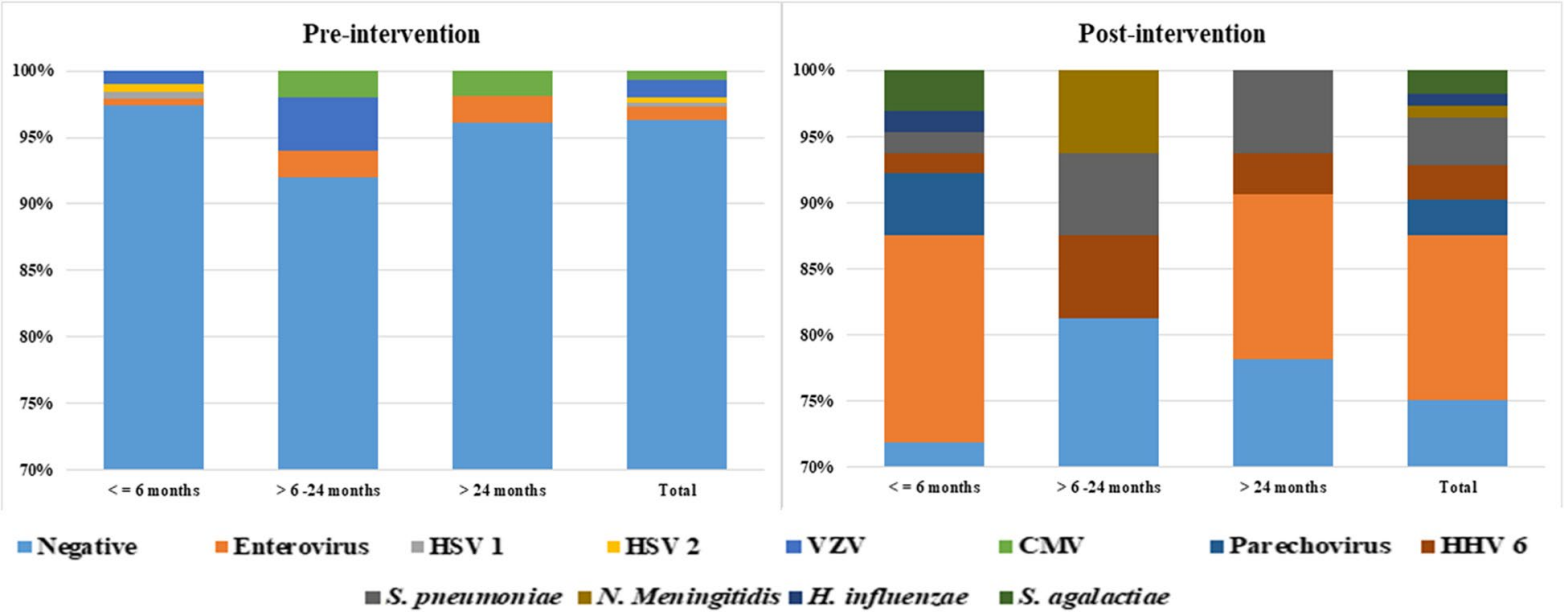
Proportion of cases of central nervous system infections caused by each pathogen in both periods according to age group

The overall positivity (any positive microbiological test) was 9.4% in the pre-intervention period and 26.8% in the post-intervention period (*p* value < 0.001). Figure 2 shows the percentage distribution of the aetiologies identified in the pre-intervention and post-intervention periods, highlighting an increase in the identification of viral aetiologies, especially enteroviruses and parechoviruses, in children under 6 months of age, along with an increase in the detection of *S. agalactiae* in the same age group. In children older than 6 months, the detection of both enterovirus and human herpesvirus 6 increased with the use of FAME; and in terms of bacteria, the identification of *S. pneumoniae* and *N. meningitidis* increased.

The CSF positivity according to the age group is compared for both periods and detailed in Table 4. In children under 6 months of age, the CSF positivity increased significantly from 2.6 to 28.1% when the use of FAMEs was incorporated. This was also observed when only CSF with altered cytology was analysed (9.7 and 42.3%). In the case of children older than 6 months of age, the same phenomenon was observed both in general positivity and in CSF, with altered cytology, both of which were statistically significant, but the difference was less than it was among children under 6 months of age.

**Table 4:** CSF positivity according to age group

	Pre-intervention	Post-intervention	***p*** value^a^
**< 6 months, n**	195	64	
**Positivity, n (%)**	5 (2.6%)	18 (28.1%)	**< 0.001**
**Participants with altered CSF, n (%)**	42 (21.5%)	27 (42.2%)	**0.005**
*Positivity in altered CSF, n (%)*	*4 (9.5%)*	*11 (40.7%)*	**0.002**
**≥ 6 months**	102	48	
**Positivity, n (%)**	6 (5.9%)	10 (20.8%)	**0.006**
**Participants with altered CSF, n (%)**	34 (33.3%)	26 (54.2)	**0.034**
*Positivity in altered CSF, n (%)*	*3 (8.8%)*	*6 (23.1%)*	0.125

^a^chi-square test; Italics: relative frequencies referred to the previous category in bold; in bold significant *p* values

During the pre-intervention period, 205/297 CSF samples had normal cytochemical fluids, and during the post-intervention period, 56/112 of the CSF samples had normal cytochemical fluids. Of these, the aetiology was identified in 1.95 and 16.07% of cases, respectively (Fig. 1), with identification mainly of viral agents (data not shown).

### Costs

The difference in total bed-days (ICU/basic bed) was not statistically significant, which is demonstrated in a marginal difference in costs. However, a significant difference in ICU bed-days was found in favour of the use of FAME (Table 5). The unit cost of this diagnostic technique (FAME) averaged $191 USD per sample in our country. In the comparison of both periods—if the ICU bed cost used for analysis is the institutional public value and the average ICU bed-cost from the private sphere—this translates into a decrease in expenses per patient between $2916 USD in public cost or $12,240 USD in private costs in favour of the period in which the use of FAME was implemented (assuming the protocols and clinical criteria for admission to the ICU of our institution are applied uniformly) (Tables 1 and 4). To this savings cost, which is viewed as a direct net cost, the value of the opportunity to use or manage the beds must be added, considering that when public hospitals in Chile do not have ICU beds available, they must purchase the services from the private sector, which makes patient care more expensive at times of high demand.

**Table 5:** Bed-day costs

	Unit bed- day costs ($USD^**d**^)	Pre-intervention		Post-intervention		Costs savings ($USD) Diff: (2) - (1)
		Quantity	Total cost ($USD) (1)	Quantity	Total cost ($USD) (2)	
**Absolute numbers**						
**N° basic bed-days**						
*Public*^*a*^	56	1908	106,848	613	34,328	72,540
*Private*^*b*^	640		1,267,200		392,320	874,880
**N° ICU bed-days**						
*Public*^*a*^	243	686	166,698	125	30,375	136,323
*Private*^*b*^	1020		699,720		127,500	572,220
**Use bed days per patient**^**c**^						
**Basic bed-days**						
*Public*^*a*^	56	7.5	420	5.8	325	95
*Private*^*b*^	640		4800		3712	1088
**ICU bed-days**						
*Public*^*a*^	243	15.5	3767	3.5	851	2916
*Private*^*b*^	1020		15,810		3570	12,240

^a^Public health insurance bed day cost

^b^Average private bed-day cost (average costs of Chilean private health institutions)

^c^Number of days in basic bed or ICU total/number of hospitalized patients in general bed or ICU

^d^Parity of chilean peso (CLP) $801.96 per dollar at 16 November 2021

## Discussion

The use of FAME significantly increases the aetiological identification of CNS infection sources [14, 22]. Even among patients with normal CSF, clinical symptoms suggestive of CNS infection were observed. Especially among patients younger than 6 months, the differences in aetiological identification of infectious agents was greater than those among older patients. Of all normal CSF collected from inpatients less than 6 months and those older than 6 months of age during the FAME period, almost 18% of infection aetiologies were identified, probably due to the ability of the FAME to recognize some viral agents, such as parechovirus, that can cause an infection without altering the CSF [23, 24]. The main aetiology of CNS infections was viral, as described in study results by S.E. Park [25]. However, the overall positivity rate in this study was 46.4%, almost double that observed in our study (24.1%), which may be explained by the difference in clinical criteria between medical teams performing lumbar punctures in children.

The increase in positive identification of the aetiology in CNS infections was the result only of the introduction of the new molecular tool, since neither the Gram stain, CSF culture nor blood cultures showed a significant difference between either period. Additionally, it is important to consider the difference in turnaround time. The use of FAME was available 24 h a day during business and non-business hours, and the processing of samples took only 65 min. Routine real-time PCR is processed only during business hours, and the response time is up to 48 h. FAME makes it possible to make timely clinical decisions [22]. During the second period evaluated, there was an increase in more complex exams.

The increase observed in imaging tests, considered an uncontrolled variable, may have been affected by events such as access to magnetic resonance imaging and tomography, as well as turnover in the team of neurology specialists who order these exams. The discharge diagnoses and aetiologies identified during the second period increased for neurologic disorders and decreased for febrile syndrome without focus. This could be due to a better diagnosis in CNS pathologies.

Goodlet et al. [26] showed 10 studies that measured the impact of FAMEs and antimicrobial use. However, there are few studies analysing the patient care costs with suspected CNS infection [27–29]. In our study, the use of FAME was associated with a significant decrease in the occupancy rate of ICU bed-days, which are the costliest beds in public hospitals and so translate into direct cost savings. This phenomenon was also observed in the study by S. Duff [27], where an overall savings per patient of USD $3481 was estimated when FAME was performed on all CSF samples obtained from patients with suspected CNS infection. This is similar to the cost savings found in our study—only we calculated this cost using the ICU bed-day concept, considering the value of public hospitals The difference between both studies lies mainly in methodology, since Duff’s study was estimated according to a mathematical model and ours was the result of the analysis of real data from the comparison of 2 cohorts, without and with the use of FAME. However, our study was conducted with a child population and thus differs from Duff’s study, which was performed using an adult population. Indirect costs should also be considered in the decreased use of ICU beds-days: decreases in children‘s exposure to the risks of being hospitalized, such as health care associated infections or adverse events, as well as biological, psychological and social factors for children and their families, also do not represent valorised savings, nor does the decrease in productivity of parents and guardians, this could represent a limitation and undervaluation of costs savings of our study. Finally, the alternative use of beds improves and is relevant to the productivity of any health institution.

Another limitation of this study is due to the retrospective design of the evaluation of the pre-intervention period, which was constructed from databases of medical care records that had already been collected. As described above, the microbiological data registry was not initially constructed with the aim of comparing etiological identification and hospitalization costs among patients with suspected CNS infection, and it is likely that not all relevant factors have been identified and recorded as antimicrobial use. Furthermore, an additional disadvantage is that many different healthcare professionals will have been involved in the patient’s care, so the measurement of variables across the entire database would likely be less accurate and consistent than that achieved in the post-intervention period that was prospective. However, the use of records that had already been collected and stored in an electronic database meant that this study was relatively inexpensive, quick and easy to perform.

Finally, molecular laboratory diagnostic tools should not be seen only as an expense or an investment of the cost centre that uses them; their value should be analysed as part of the overall cost of the institution, taking into consideration the entire care process. In times of high demand for beds in our country, the public health system buys bed-days from the private system; a diagnostic tool like FAME enables the optimisation of resources in health care.

## Conclusions

FAME use significantly increases the aetiological identification of infectious agents in children with suspected CNS infections, in both those with or without altered cytochemical CSF. This increase is greater among children under 6 months of age. FAME use enables more accurate aetiological identification and timely diagnosis, and it results in better opportunities for adequate treatment to be provided and for better clinical outcomes to be achieved [30]. The cost/benefits ratio between FAME test value and ICU bed-day cost is favourable to the use of FAME because the investment in the protocoled implementation of this test effectively implies a significant cost savings for the institution, at least in terms of ICU bed-days as direct cost. It is important to emphasize that there are indirect savings associated with patient safety, as well, including the reduction of psychosocial risks for the child and a reduced impact on the economic productivity of parents or guardians. Additionally, the alternative use of beds thus enabled is relevant to the management of the health care institution. We recommend the use of this diagnostic tool for the study of CSF samples from the age group of under 6 months or from those requiring admission to intensive care. Implementation of FAME in Chilean public hospitals in the diagnosis of CNS infection saves public resources and improves the opportunity for effective treatment based on accurate aetiological diagnoses of infectious agents.

## Data Availability

The datasets used and/or analysed for the current study are available from the corresponding author upon reasonable request. It is not possible to deposit the database publicly due to ethical reasons and protection of patient data according to Chilean law No. 20584, however, if these are required for review by the journal or to be part of a publication with data from different countries, the database will be shared duly encrypted.

## Abbreviations

CNS: Central Nervous System
FAME: FilmArray® Meningoencephalitis
ABM: Acute Bacterial Meningitis
CSF: Cerebrospinal Fluid
CLSI: Clinical and Laboratory Standards Institute
PCR: Polymerase Chain Reaction
ED: Emergency Department
CMV: Cytomegalovirus
HSV-1: Herpes simplex virus 1
HSV-2: Herpes simplex virus 2
HHV-6: Human herpes virus
HpeV: Human parechovirus
VZV: Varicella zoster virus
EBV: Epstein Barr virus
DNA: Deoxyribonucleic acid
ICU: Intensive Care Unit
PIM: Paediatric Index of Mortality
EEG: Electroencephalogram
ID: Identification
IQR: Interquartile Range
Diff: Difference
